# Anti-Obesity and Anti-Hyperglycemic Effects of *Meretrix lusoria* Protamex Hydrolysate in *ob/ob* Mice

**DOI:** 10.3390/ijms23074015

**Published:** 2022-04-05

**Authors:** Min Ju Kim, Ramakrishna Chilakala, Hee Geun Jo, Seung-Jae Lee, Dong-Sung Lee, Sun Hee Cheong

**Affiliations:** 1 Department of Marine Bio-Food Sciences, College of Fisheries and Ocean Sciences, Chonnam National University, Yeosu 59626, Korea; modori96k@naver.com (M.J.K.); ramach2006@gmail.com (R.C.); altkwh@naver.com (H.G.J.); 2 Immunoregulatory Material Research Center, Korea Research Institute of Bioscience and Biotechnology (KRIBB), Jeongeup 56212, Korea; seung99@kribb.re.kr; 3 Department of Pharmacy, College of Pharmacy, Chosun University, Dong-gu, Gwangju 61452, Korea; dslee2771@chosun.ac.kr

**Keywords:** *Meretrix lusoria* protamex hydrolysate, anti-obesity, anti-hyperglycemic, *ob*/*ob* mice

## Abstract

*Meretrix lusoria* (*M. lusoria*) is an economically important shellfish which is widely distributed in South Eastern Asia that contains bioactive peptides, proteins, and enzymes. In the present study, the extracted meat content of *M. lusoria* was enzymatic hydrolyzed using four different commercial proteases (neutrase, protamex, alcalase, and flavourzyme). Among the enzymatic hydrolysates, *M. lusoria* protamex hydrolysate (MLPH) fraction with MW ≤ 1 kDa exhibited the highest free radical scavenging ability. The MLPH fraction was further purified and an amino acid sequence (KDLEL, 617.35 Da) was identified by LC-MS/MS analysis. The purpose of this study was to investigate the anti-obesity and anti-hyperglycemic effects of MLPH containing antioxidant peptides using *ob*/*ob* mice. Treatment with MLPH for 6 weeks reduced body and organ weight and ameliorated the effects of hepatic steatosis and epididymal fat, including a constructive effect on hepatic and serum marker parameters. Moreover, hepatic antioxidant enzyme activities were upregulated and impaired glucose tolerance was improved in obese control mice. In addition, MLPH treatment markedly suppressed mRNA expression related to lipogenesis and hyperglycemia through activation of AMPK phosphorylation. These findings suggest that MLPH has anti-obesity and anti-hyperglycemic potential and could be effectively applied as a functional food ingredient or pharmaceutical.

## 1. Introduction

Obesity is a disorder caused by the metabolism of carbohydrates and fats that results in excessive fat accumulation in the liver and adipose tissues [1]. It is a serious disorder accompanied by various chronic diseases including diabetes, insulin resistance, and hepatic steatosis [2,3]. Cellular oxidative stress can generate excessive reactive oxygen species (ROS) and alter antioxidant capacity, which can trigger proinflammatory signaling pathways, consequently modulating the gene expression levels of several proinflammatory cytokines [4]. It is known that cytokines such as tumor necrosis factor-α (TNF-α) and monocyte chemoattractant protein-1 (MCP-1) in adipocytes are positively correlated with obesity [5,6]. Enzymes such as sterol regulatory element-binding proteins (SREBPs), fatty acid synthase (FAS), and acetyl-CoA carboxylase (ACC) are responsible for the synthesis of fatty acids and cholesterol [7].

Due to a point mutation of the leptin gene, *ob*/*ob* mice are known to develop obesity, glucose intolerance, insulin resistance, and fatty liver [8]. Fatty livers of *ob*/*ob* mice show accumulation of triglycerides due to increased lipogenesis in parallel with elevated gene expression of lipogenic enzymes such as FAS and ACC by regulating SREBP transcription [9]. The adenosine monophosphate-activated protein kinase (AMPK) enzyme can act as an energy homeostasis sensor. It is a key factor in suppressing obesity by regulating adipocyte lipolysis, hepatic lipid metabolism, fatty acid oxidation in skeletal muscle, and glucose transport [10]. Phosphorylated AMPK (pAMPK) enzyme can suppress the synthesis of fatty acids in the liver and adipose tissues and restore cellular energy homeostasis [11,12]. Hence, regulating AMPK expression and activation is important to prevent obesity and related complications [13].

Over the years, many anti-obesity drugs have been available, but most have since been withdrawn from the market due to their serious adverse effects. Anti-obesity drugs such as dexfenfluramine and fenfluramine were withdrawn due to their cardiac side effects, and several anti-obesity drugs were withdrawn by the European Medicines Agency in 2000 [14]. Natural products have attracted growing interest in the development of anti-obesity drugs because they are less toxic than synthetic drugs and have fewer side effects. In particular, animal tissues contain various bioactive components which have anti-obesity and anti-diabetic effects, thus there is a growing interest in using them to treat or prevent many human diseases [15]. Recently, Kang et al. [16] identified potential antioxidants from the proteins of sea snails (*Turbo cornutus*), comprising nine distinct low molecular bioactive peptides with H_2_O_2_ scavenging ability, suggesting that *T. cornutus* might be employed as a functional food for human health. Wang et al. [17] demonstrated that herring milt enzymatic protein hydrolysate (HMPH) and dried ground herring milt products (HMDP) could provide beneficial effects in high-fat-diet-induced obese mice. Herring milt treatment can significantly improve insulin resistance and glucose homeostasis. Kim et al. [18] also reported that yellow catfish protein hydrolysate treatment had anti-obesity and ameliorating effects by regulating hepatic glucose enzymes and antioxidant activities in mice fed a high-fat diet. Most peptides prevent obesity and diabetes by regulating the neuroendocrine system [19], although bioactive peptides have poor chemical stability and short-term effectiveness [20]. Peptide isolates have been used in formulations for the production of desired qualities in functional foods. Encapsulation is the method of incorporating bioactive peptides with other compounds as a wall material to improve their stability [21]. Choi et al. [22] developed a new formulation method using a double emulsion system (W_1_/O/W_2_) containing bioactive peptides to improve their physical and chemical stability. In addition, some formulation technologies, such as nanotechnology, coating, and microcapsule methods, can also be used to improve stability [20].

Shellfish are widely grown and cultivated as an important food source in Japan, Korea, and China [23,24]. *M. lusoria* (hard clam) is the most abundant bivalve in South-East Asia. It is an excellent source of bioactive components, including proteins, peptides, polysaccharides, enzymes, minerals, essential amino acids, vitamins, and fatty acids, especially *n*−3 polyunsaturated fatty acids including apoptotic-inducing epidioxysterols, which are all beneficial for health [25]. Furthermore, *M. lusoria* protein hydrolysates contain numerous bioactive compounds which have been isolated by enzymatic hydrolysis using proteases [26]. The degree of proteolytic enzymes hydrolysis has a direct impact on the length and size of enzymatic hydrolysates containing peptides. It seems that protamex can improve the solubility of protein and fatty acid compositions, and recover peptides and unsaturated fatty acids. When compared to alcalase and flavourzyme, protamex produces a hydrolysate with lower turbidity and better thermal stability. Enzymatic hydrolysis is one of the most efficient methods for recovering bioactive compounds without nutrient loss, thus increasing the commercial value. It has been reported that novel peptides derived from oyster enzymatic hydrolysates have several biological benefits, including anti-cancer and angiotensin-converting enzyme inhibitory effects [27,28]. However, to the best of our knowledge, there have been no reports about the anti-obesity and anti-hyperglycemic effects of enzymatic hydrolysate from *M. lusoria*. Therefore, the objective of the present study was to evaluate antioxidant activities of enzymatic hydrolysates from *M. lusoria* prepared with four commercial proteases (neutrase, protamex, alcalase, and flavourzyme) and identify amino acid sequences of purified peptides from MLPH with the strongest free radical scavenging abilities. To further investigate the anti-obesity and anti-hyperglycemic potential of MLPH containing antioxidant peptide, the effect of MLPH in *ob*/*ob* mice were also investigated. This is the first report on the anti-obesity and anti-hyperglycemic effects of enzymatic hydrolysates derived from *M. lusoria*.

## 2. Results

### 2.1. M. lusoria Enzymatic Hydrolysate Yield (%) and the Antioxidant Activity of Their Fractions

Hydrolysates were obtained from *M. lusoria* using hydrolysis enzymes. The yield was around 64% to 65% using neutrase and protamex, and around 59% with alcalase and flavourzyme. However, almost all enzymatic hydrolysates had a much higher than 50% yield. The antioxidant activity of neutrase, protamex, alcalase, and flavourzyme hydrolysates, based on DPPH and ABTS radical scavenging assays (IC_50_ values), is shown in Table 1. The results revealed that hydrolysates with molecular weight (MW) ≤ 1 kDa or 1–3 kDa presented the highest DPPH and ABTS radical scavenging activity. These ≤1 and 1–3 kDa fractions have plenty of amino acid groups that can more readily donate electrons to free radicals (DPPH and ABTS) than larger peptides (3–5 and ≥5 kDa). Fractions with MW ≤ 1 kDa from all four hydrolysates showed the highest DPPH and ABTS radical scavenging activity. Although the neutrase enzymatic hydrolysate fraction showed a higher yield percentage (65.2%), it showed a higher IC_50_ value for ≤1 kDa fraction (2.67) with lower scavenging activity for ABTS radicals. The protamex yield was also high (64.1%), and its ≤1 kDa hydrolysate fraction showed a lower IC_50_ value (1.38–1.58), with higher scavenging activity for both DPPH and ABTS radicals. Then protamex ≤1 kDa hydrolysate fraction was further purified and the peptide sequencing was identified by liquid chromatography tandem mass spectrometry (LC-MS/MS) analysis. Subsequently, the ≤1 kDa *M. lusoria* protamex hydrolysate (MLPH) fraction was chosen for further treatment of *ob*/*ob* mice due to its high DPPH and ABTS radical scavenging activity.

### 2.2. MLPH (≤1 kDa) Fraction Purification and Peptide Sequence Identification

**Figure 1:**
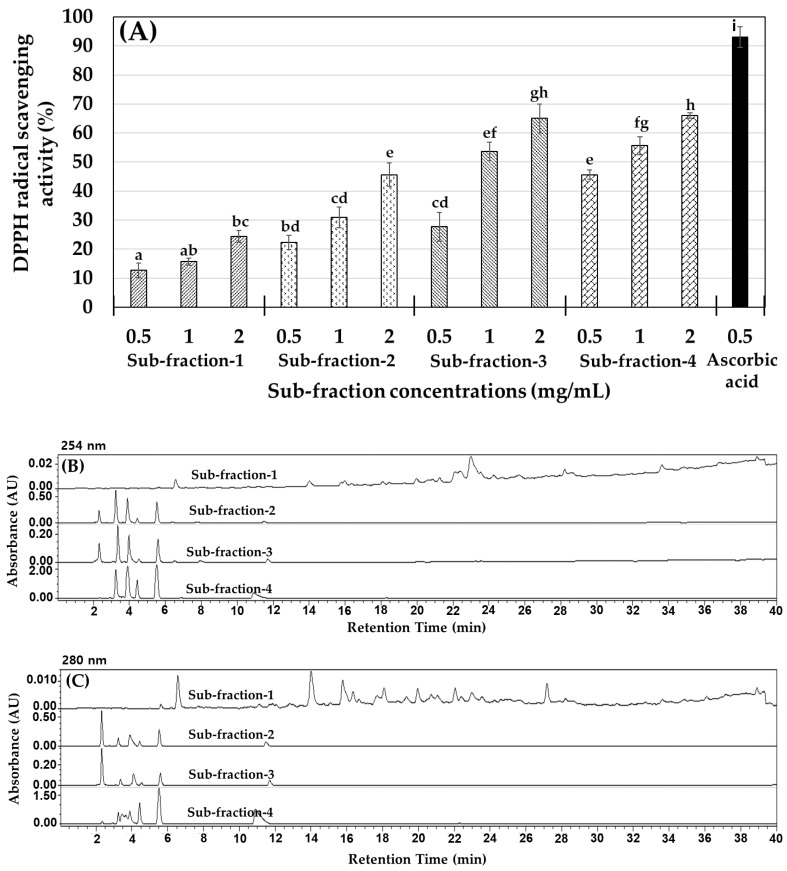
*M. lusoria* protamex hydrolysate (MLPH): (**A**) DPPH radical scavenging activity of all sub-fractions, (**B**,**C**) RP-HPLC patterns of sub-fractions monitored at 254 nm and 280 nm, respectively. Each value represents the mean ± SEM in triplicate (*n* = 3). ^a–i^ Values not sharing a common letter are significantly different at *p* < 0.05 by Tukey’s multiple comparison test.

Considering its antioxidant effect on DPPH and ABTS radical scavenging activity, the protamex hydrolysate (≤1 kDa) fraction was employed for purification and identification. In these processes, the MLPH fraction (≤1 kDa) was separated into four sub-fractions (1 to 4) according to molecular size using a Sephadex G-25 chromatography column, and monitored at 254 and 280 nm absorbance. The obtained sub-fraction 1 (0.5 mg), sub-fraction 2 (397.6 mg), sub-fraction 3 (4452.7 mg), and sub-fraction 4 (139.6 mg) were collected and their HPLC patterns and DPPH radical scavenging activity were measured, as shown in Figure 1. The results in Figure 1B,C indicate that sub-fractions 2 to 4 had similar patterns. Among them, sub-fraction 3 had the highest free radical scavenging ability (Figure 1A) with high yield (%), as determined by size exclusion chromatography (SEC). Sub-fraction 3 was purified using a YMC Triart C18 column, and then a peptide sequence analysis was performed by mass spectroscopy (MS/MS) analysis.

**Figure 2:**
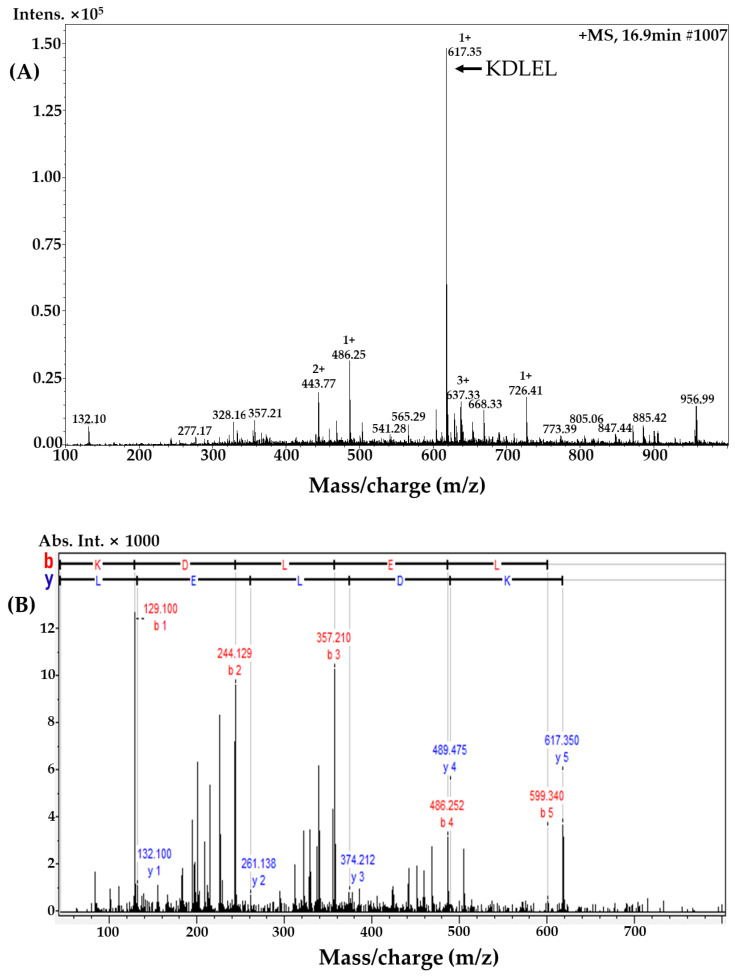
Molecular mass and amino acid sequence identification using sub-fraction 3 from MLPH fraction: (**A**) liquid chromatography–mass spectrometry; (**B**) tandem mass spectrometry (MS/MS) spectrum (*m*/*z* 617.35).

The purified sub-fraction 3 was determined by a Micro Q-TOF III ESI mass spectrometer. Based on this approach, one major signal was identified as KDLEL (Lys-Asp-Leu-Glu-Leu), as shown in Figure 2. The selective ion chromatogram of KDLEL (*m*/*z* 617.35, t_R_: 16.9 min), shown in Figure 2A, indicated that this was the major peptide in this fraction. The MS/MS spectrum of *m*/*z* 617.35 in which b and y series ions were matched to the predicted peptide sequence of KDLEL is shown in Figure 2B. Lee and Byun [29] suggested that small peptides containing 2 to 20 amino acids have greater antioxidant activity compared to larger peptides.

### 2.3. Metabolic Characteristics of ob/ob Mice Fed a Standard Diet and Mice Administered MLPH

We investigated the in vivo effect of MLPH fraction for 6 weeks using normal mice and *ob*/*ob* mice as an obese animal model. The food efficiency ratio, body weight, organ weight, and total fat content results are shown in Table 2. It was found that *ob*/*ob* mice in the CON and MLPH groups showed no significant difference in food intake, feed efficiency ratio (FER), or weight gain after 6 weeks of treatment, although, the NOR group (C57BL/6J mice) was significantly different from the CON and MLPH groups of *ob*/*ob* mice. Heart and kidney weights showed significant differences between the NOR and CON groups. In the MLPH treatment groups, heart and kidney weight were reduced, but this was not significantly different to those of the NOR group. In addition, the gastrocnemius muscle showed no significant differences among all experimental groups, while, liver weight showed a significant difference between the NOR and CON groups. MLPH treatment groups showed reduced liver weight compared to the CON group. A fat content analysis indicated that mesenteric fat weight was high in the CON group, i.e., significantly higher than in the MLPH groups. Similar results were found for epididymal and kidney fat content. On the other hand, retroperitoneal fat content was not significantly different between the CON and MLPH groups. Total fat content was also high in the CON group and was significantly decreased after MLPH fraction treatment at low or high doses. However, total fat content in the NOR group was significantly different compared to the CON and MLPH groups of *ob*/*ob* mice.

### 2.4. Effect of MLPH Fraction on Impaired Glucose Tolerance in ob/ob Mice

**Figure 3:**
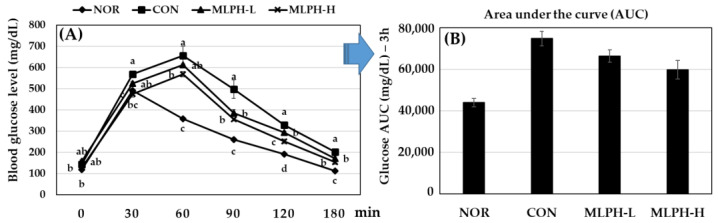
(**A**) Glucose tolerance test and (**B**) area under the curve results for mice treated with MLPH fractions for 6 weeks and fasted for 14 h before intraperitoneal injection of glucose (2 g/kg body weight). After glucose injection, blood samples were collected from the tail vein at 0, 30, 60, 90, 120, and 180 min. Each value represents the mean ± SEM (*n* = 6). ^a–c^ Values not sharing a common letter are significantly different at *p* < 0.05 by Tukey’s multiple comparison test.

After 6 weeks of treatment with MLPH at a low (150 mg) or high (300 mg) dose, glucose-induced (2 g/kg body weight) diabetic *ob*/*ob* mice were subjected to a glucose tolerance test. The results, shown in Figure 3, reveal that blood glucose levels were significantly suppressed after 30 min in the normal group and after 60 min in the CON and MLPH treated groups. Moreover, the MLPH groups treated with protamex extract showed drastically decreased blood glucose levels when compared to the CON group. Blood glucose levels were suppressed from 8 to 22% within 30 to 90 min in the MLPH-L group, and from 16 to 28% within 30 to 90 min in the MLPH-H group when compared to the CON group, which showed significantly different levels.

### 2.5. Hepatic Histological Changes and Marker Enzymes, including Antihyperlipidemic Effect, in MLPH treated ob/ob Mice

**Figure 4:**
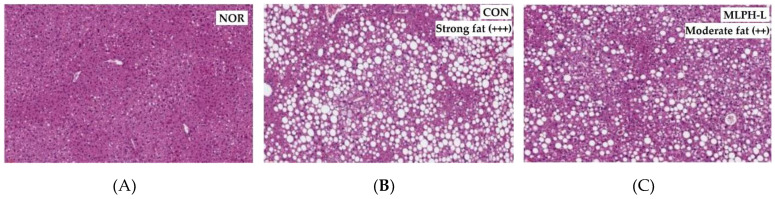

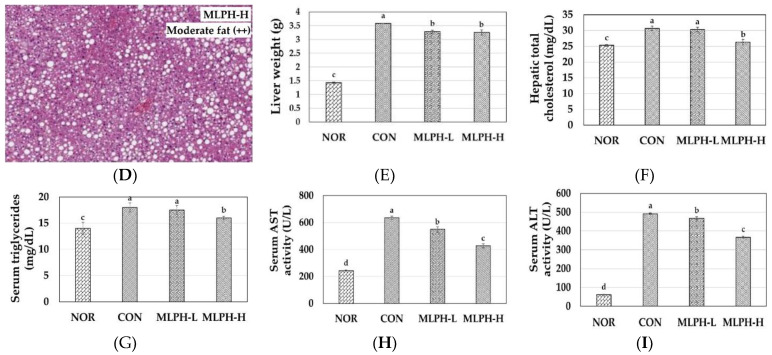
Effect of MLPH fraction on histology of mouse liver in (**A**) NOR, (**B**) CON, (**C**) MLPH-L, and (**D**) MLPH-H groups; (**E**) liver weight; (**F**) hepatic total cholesterol, including hepatic marker enzymes; (**G**) serum triglycerides; (**H**) serum AST; and (**I**) serum ALT. Each value represents the mean ± SEM (*n* = 6). ^a–c^ Values not sharing a common letter are significantly different at *p* < 0.05 by Tukey’s multiple comparison test.

The results regarding the effect of MLPH fraction on liver histological changes and other parameters are shown in Figure 4. Liver histology results using hematoxylin and eosin (H&E) staining, shown in Figure 4A–D, indicate that *ob*/*ob* mice in the CON group had more severe macrovesicular steatosis than those in the NOR group. Moreover, liver weight was increased with strong fat (+++) deposition (Figure 4b) in the CON group due to a substantial accumulation of hepatic lipid droplets. The CON group also showed a high food efficiency ratio (Table 2), which causes obesity. It is known that *ob*/*ob* mice possess a recessive mutation in the leptin gene, and hence, abnormal leptin protein, leading to more food intake and body weight gain than normal mice. However, MLPH treatment decreased macrovesicular steatosis in both MLPH-L and MLPH-H groups (Figure 4C,D). It also decreased fat deposition, with moderate (++) fatty levels observed in liver tissues. Liver weight in the MLPH groups was slightly but significantly reduced compared to the CON group (Figure 4E). Hepatic total cholesterol (Tch) and concentrations of serum triglycerides levels were significantly reduced in the MLPH-H group when compared to the CON and MLPH-L groups (Figure 4F,G). The activity of marker enzymes (AST and ALT) was significantly reduced in MLPH groups when compare to the CON group (Figure 4H,I). Meanwhile, hepatic triglycerides, serum LDL and HDL cholesterol were not significantly different when compared to the CON group, as shown in Figure S1 as a Supplementary File.

### 2.6. Epididymal Fat Changes and Metabolic Variables in MLPH Treated ob/ob Mice

**Figure 5:**
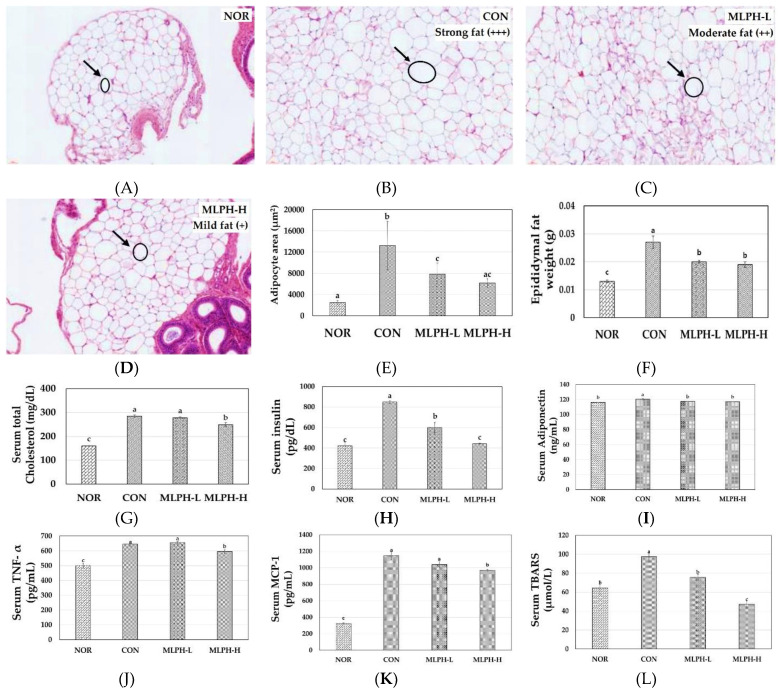
Effects of MLPH fraction on epididymal fat in (**A**) NOR, (**B**) CON, (**C**) MLPH-L, and (**D**) MLPH-H groups; (**E**) adipocyte area; (**F**) epididymal fat weight; metabolic parameters: (**G**) serum total cholesterol, (**H**) serum insulin, (**I**) serum adiponectin, (**J**) serum TNF-α cytokine, (**K**) serum MCP-1 cytokine, (**L**) serum TBARS levels. Each value represents the mean ± SEM (*n* = 6). ^a–c^ Values not sharing a common letter are significantly different at *p* < 0.05 by Tukey’s multiple comparison test.

To investigate the anti-obesity effect of MLPH extract on epididymal fat, H&E staining was performed. The results, including metabolic characteristics, are shown in Figure 5. Histological changes of epididymal tissues indicated that the adipocytes were much larger in the CON obesity group (*ob*/*ob* mice) than in the NOR group (C57BL/6J mice). However, the adipocytes in groups of *ob*/*ob* mice treated with MLPH (at low and high doses) were drastically smaller than those of the CON group (Figure 5A–D). The total adipocyte area also increased in the CON group when compared to the NOR group, while with MLPH treatment, the total adipocyte area was significantly decreased in a dose dependent manner (Figure 5E). Moreover, epididymal tissue weight was increased (Figure 5F), with strong (+++) fat deposition in the CON group due to the high intake of food compared to the NOR group (Table 2). However, moderate (++) and mild (+) fat deposition occurred in the MLPH-L and MLPH-H groups, respectively. In addition, we analyzed obesity-associated serum parameters to confirm the anti-obesity property of the MLPH extract. Serum total cholesterol, insulin, adiponectin, tumor necrosis factor α (TNF-α), monocyte chemoattractant protein-1 (MCP-1), and TBARS levels were increased in the CON group compared to the NOR group. However, these increases were significantly suppressed with MLPH fraction treatment (Figure 5G–L). The animal body weight, serum protein, and albumin levels were significantly elevated in the CON group compared to the NOR group. Meanwhile these parameters were not significantly different in MLPH treatment when compared to the CON group. Blood urea nitrogen (BUN) and creatinine levels in the NOR group were not significantly different compared to the other experimental *ob*/*ob* groups, as shown in Figure S2 as a Supplementary File.

### 2.7. Hepatic Antioxidant Enzyme Activity in Normal, Control, and MLPH Treated Mice

To evaluate the ability of enzymatic MLPH extract to prevent hepatic impairment in obese mice, GST, GPx, GR, SOD, and CAT enzyme activity and GSH levels were analyzed using liver homogenates. The results are shown in Table 3. It was revealed that hepatic GSH levels and GST, GPx, GR, SOD, and CAT enzyme activity were decreased in the CON group compared to the NOR group. However, enzyme extract treatment for 6 weeks (MLPH groups) increased the GSH levels and antioxidant enzyme activity in a dose-dependent manner, although GST activity in the CON group was not significantly different from that in the MLPH-L or MLPH-H groups. On the other hand, GST enzyme activity tended to increase with the increasing concentration of enzymatic hydrolysate used.

### 2.8. Effects of MLPH Fraction on AMPK, SREBP, FAS, and ACC Protein Expression in Control Obese Mice

**Figure 6:**
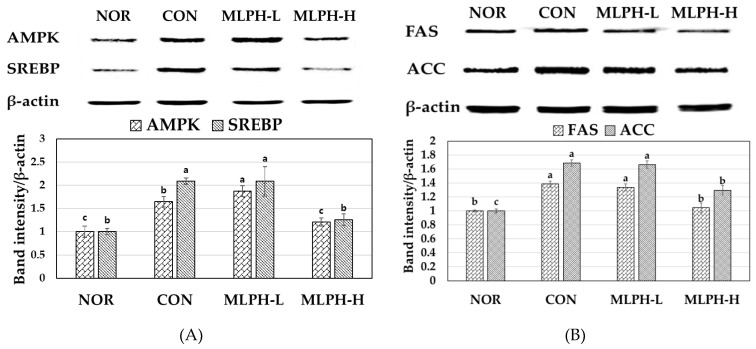
Effects of MLPH extract on protein expression levels of (**A**) AMPK and SREBP, and (**B**) FAS and ACC in liver tissue of normal and experimental *ob*/*ob* mice. Each value represents the mean ± SEM (*n* = 6). ^a–c^ Values not sharing a common letter are significantly different at *p* < 0.05 by Tukey’s multiple comparison test.

We examined protein expression levels of AMPK, SREBP, FAS, and ACC by western blot analysis using MLPH to treat obese mice. The results are shown in Figure 6. It was found that the expression levels of AMPK, SREBP, ACC, and FAS were upregulated in the livers of *ob*/*ob* mice in the CON group. After 6 weeks of MLPH treatment, these levels were significantly reduced by MLPH in a dose-dependent manner; these results were close to those of the NOR group. This trend provides indirect evidence that MLPH has an anti-obesity effect by suppressing fatty acid synthesis in *ob*/*ob* mice.

### 2.9. Effects of MLPH on Hepatic mRNA Expression Levels Related to Lipogenesis and Gluconeogenesis in Obese Mice

**Figure 7:**
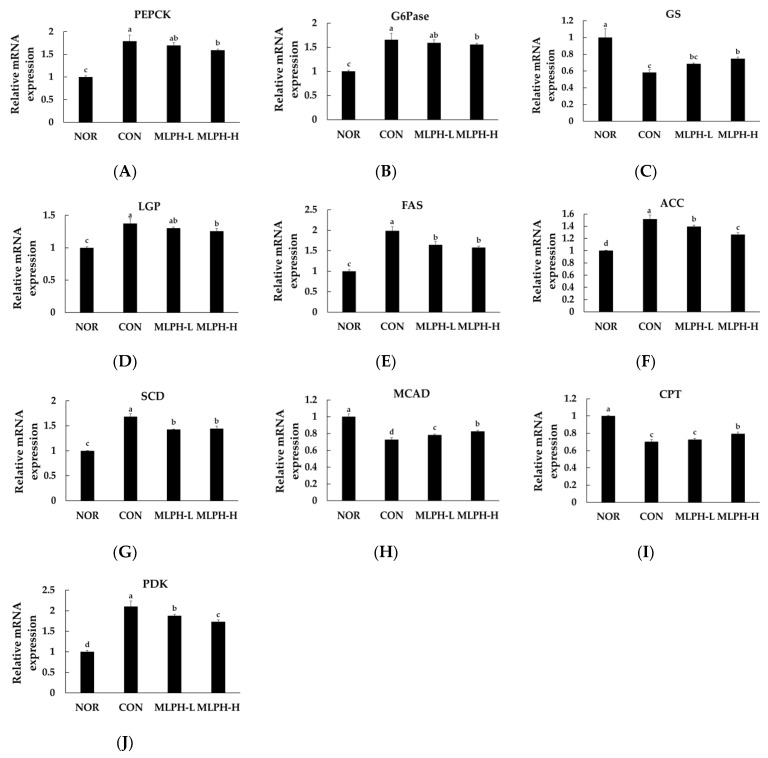
Effects of MLPH on mRNA expression of lipid and glucose metabolism related genes: (**A**) phosphoenolpyruvate carboxykinase (PEPCK), (**B**) G6Pase, (**C**) glycogen synthase (GS), (**D**) liver glycogen phosphorylase (LGP), (**E**) fatty acid synthase (FAS), (**F**) acetyl-CoA carboxylase (ACC), (**G**) stearoyl-CoA desaturase (SCD), (**H**) medium chain acyl-CoA dehydrogenase (MCAD), (**I**) carnitine palmitoyltransferase (CPT), and (**J**) pyruvate dehydrogenase kinase (PDK). Each value represents the mean ± SEM (*n* = 6). ^a–c^ Values not sharing a common letter are significantly different at *p* < 0.05 by Tukey’s multiple comparison test.

The hepatic expression of mRNAs related to lipogenesis and gluconeogenesis enzymes in *ob*/*ob* mice was evaluated. The results showed that the levels of PEPCK and G6Pase enzyme expression were upregulated in control obese mice, indicating an accumulation of hepatic glycogen and circulating glucose levels. PEPCK and G6Pase are well known as gluconeogenic enzymes that function in liver tissues. After treatment with MLPH, their expression levels were significantly (*p* < 0.05) downregulated in obese mice in a dose-dependent manner (Figure 7A,B). Quantitative mRNA expression of glycogen synthase (GS) was decreased and glycogen phosphorylase (GP) expression was increased in liver tissues of the control group of obese mice. These results suggest that decreased GS levels and elevated GP levels in the liver of obese mice are responsible for diminished glycogen synthesis. This also confirms that obesity is associated with insulin resistance, thus favoring the development of diabetes. In MLPH groups, GS levels were increased significantly, whereas LPG levels were decreased (Figure 7C,D). Moreover, the expression of lipogenic enzymes FAS, ACC, and SCD was elevated in control obese mice, while MLPH treatment significantly suppressed hepatic mRNA expression (Figure 7E–G). The expression of fatty acid oxidation related genes of MCAD and CPT mRNA was decreased in the control obese group compared to the NOR group. However, mRNA expression was significantly increased by MLPH treatment in a dose-dependent manner (Figure 7H,I). Moreover, in obese mice, elevated PDK enzyme expression was significantly decreased by MLPH (Figure 7J).

## 3. Discussion

In this study, we demonstrated that MLPH had anti-obesity and anti-hyperglycemic effects in an *ob*/*ob* mouse model. The antioxidant activity of hydrolysates depends on their molecular weight distribution [30]. In this study, protamex, alcalase, and flavourzyme hydrolysates from *M. lusoria* with low-molecular-weight peptides (≤1 kDa) were found to have lower IC_50_ values, corresponding to higher scavenging activity for DPPH and ABST radicals. Moreover, an amino acid sequence (Lys-Asp-Leu-Glu-Leu) was identified in the protamex hydrolysate sub-fraction with an IC_50_ value of less than 1.38 mg/mL and low molecular weight (617.35). Similarly, nine low molecular bioactive peptides were identified in *Turbo cornutus* protamex hydrolysate with IC_50_ values of 0.6 to 7.2 mg/mL [15]. However, MLPH containing KDLEL (Lys-Asp-Leu-Glu-Leu) fraction was shown to be rich in lysine, aspartic acid, leucine, and glutamic acids, which have a greater free radical scavenging effect. Leucine is a hydrophobic amino acid which is essential for accessibility to hydrophobic targets. Leucine may inhibit lipid peroxidation by increasing the solubility of lipid-containing peptide molecules and facilitating better interactions for free radicals [29]. He et al. [31] also used various proteases to obtain rapeseed protein hydrolysate fractions of different molecular sizes to improve DPPH and superoxide scavenging properties. Our results suggest that *M. lusoria* hydrolysates ≤1 kDa in size could be used as natural antioxidants to prevent obesity and diabetes due to their lower IC_50_ value and rich leucine content, and as a functional food to improve antioxidant properties.

In the current study, we used hydrolysates of protamex extraction ≤1 kDa in size for further studies due to their high yield and antioxidant activities. Treatment with MLPH fraction improved the diminished glucose tolerance in *ob*/*ob* mice, proving that this enzymatic extract has anti-hyperglycemic activity. Moreover, a high dose can improve the suppressive effect on blood glucose levels in obese diabetic animals. Similar results were observed by Choi et al. [32], who reported melanian snail protein hydrolysates with anti-diabetic effect against liver damage. However, the physiological and molecular mechanisms of MLPH fractions in fat and glucose metabolism have not yet been determined.

Obesity and related metabolic disorders, such as hyperlipidemia and weight gain, are major causes of the development of diabetes and cardiovascular diseases [33]. The current study indicates that the CON *ob*/*ob* mice exhibited significantly improved body weight gain, including liver weight. The histology of liver tissues from control obese mice showed microvesicular steatosis, hepatocellular bloating, and vacuole formation with fat accumulation. Fatty liver due to elevated metabolic markers such as total cholesterol, AST, ALT, and triglycerides in CON *ob*/*ob* mice is an indication of increasing hepatic steatosis [34]. Garces-Rimon et al. [35] reported that pepsin egg white hydrolysate with small peptide chain effectively prevented hepatic steatosis and reduced oxidative stress in Zucker fatty rats. Thus, our results suggest that *M. lusoria* containing antioxidant oligopeptide could be used as a promising nutritive supplement to treat hepatic steatosis.

Adipose tissue is an active endocrine organ secreting a variety of adipokine hormones that can mediate the metabolic and inflammatory consequences of obesity [36]. High serum levels of adiponectin, TNF-α, and MCP-1 are considered as major causes of obesity-associated chronic inflammation in CON *ob*/*ob* mice [37]. The current study indicates epididymal tissue weight gain by strong fat deposition with larger adipocytes in control obese mice, with concurrently increased body weight compared to normal mice. The increased serum adiponectin level in control *ob*/*ob* mice can lead to obesity due to reduced energy consumption. However, the serum adiponectin levels in MLPH treated groups were similar to those of the NOR group.

TNF-α is a major protein related to obesity that plays an important role in regulating fat metabolism; it is positively correlated with obesity and inhibits intracellular signaling from the insulin receptor, leading to diabetes [38]. Increased expression of TNF-α in adipose and liver tissues is characterized by high-intensity inflammation. Secretion of TNF-α is also correlated with cell size [39]. Increasing serum MCP-1 levels indicate the development of pathogenic obesity in CON *ob*/*ob* mice [40]. In addition, elevated MCP-1 levels initiate the infiltration of macrophages in adipose tissue and increase insulin resistance, thus facilitating the development of pre-diabetes [40,41]. Elevated MCP-1 levels are a known factor in diabetic patients and are known to contribute to the development of diabetes complications [41]. Elevated MCP-1 may also be responsible for the development of hepatic steatosis and increased serum triglyceride concentrations [42,43]. TBARS concentrations are also increased in obese mice and are considered to be early biomarkers of oxidative damage [44,45,46]. In the current study, TNF-α, MCP-1 and TBARS levels were elevated in the CON group, increasing the likelihood that *ob*/*ob* mice would develop obesity along with pre-diabetic condition and confirming the anti-obesity effect of MLPH by reducing TNF-α, MCP-1 and TBARS levels, although the mechanism of improvement of adipose tissue by MLPH treatment was not clearly revealed. In obese mice, de-Medeiros et al. [47] found that tiny hydrolyzed proteins and vegetable peptides efficiently reduced inflammatory proteins such as TNF-α and MCP-1. Similarly, the current study found that MLPH containing a short peptide chain could significantly modulate serum levels of adiponectin, TNF-α, MCP-1, and TBARS in *ob*/*ob* mice.

Obesity is closely correlated with insulin resistance [48], and increased adipocyte size indicates reduced insulin sensitivity [49]. Hyperinsulinemia is a well-established marker of insulin resistance [50] in patients with obesity and pre-diabetes who may develop permanent diabetes in the future. Panag et al. [51] demonstrated that the fasting serum insulin level is a very specific and sensitive marker for assessing insulin resistance. They reported that the fasting insulin level could be used as an easy test to detect insulin resistance in patients with obesity. They found that fasting serum insulin levels were elevated in obese animals. Serum glucose levels and incidence of diabetes were also increased in obese animals. It is evident from our results that there was a significant increase in the level of serum insulin in control obese mice, and that MLPH treatment reduced serum insulin levels and the pre-diabetic condition. Similarly, improved insulin sensitivity was observed in high-fat-diet-induced obesity mice by treatment with herring milt protein hydrolysate [16].

In the CON group of obese mice, there was pro-oxidant status, because visceral adipose tissue can secrete proinflammatory cytokines [52] and reduce antioxidant enzyme activity [53]. This decrease in antioxidant enzyme activity might have been due to exhaustion and rapid consumption of stored antioxidant enzymes in the process of fighting against the free radical generation in obesity. Free radical generation contributes by involving mechanisms of obesity-associated oxidant stress and increased fat deposition in organs [4]. In addition, hyperglycemia, hypertension, and hypercholesterolemia with high levels of lipid peroxidation are also possible causes of increased oxidant stress in the obese state [54]. Harmful reactive oxygen species are generated along with elevated lipid peroxidation in control *ob*/*ob* mice, leading to organ damage [55,56]. In the present study, it was indicated that the levels of antioxidant enzymes GST, GPx, GR, SOD, CAT, and GSH in CON obese (*ob*/*ob*) mice declined due to increased oxidative stress and inflammation in the liver tissue. The activity of these enzymes was elevated with MLPH treatment in a dose-dependent manner. Santos-Sanchez et al. [57] also reported that treatment with *Lupinus angustifolius* protein hydrolysates boosted hepatic total antioxidant capacity, and then decreased hepatic inflammation and abdominal obesity in western diet-fed-apoE-/- mice. In the present study, the degradation of hepatic antioxidant enzyme activity in the CON group was effectively inhibited by MLPH treatment, suggesting that MLPH treatment is favorable to increase antioxidant effects in *ob*/*ob* mice.

Excessive fat accumulation in the liver and adipose tissues can lead to hyperlipidemia and obesity [58]. In obese mice, AMPK activity is dysregulated or inactivated due to increasing pathogenic metabolic disorders [59]. Depleted AMPK activity and other metabolic disorders are associated with obese mice, leading to high insulin resistance [60]. The current results suggest that impaired AMPK phosphorylation is activated by MLPH, thus increasing AMPK-activated phosphorylation (p-AMPK) activity, inhibiting acetyl-CoA carboxylase and malonyl-CoA levels, and enhancing fatty acid oxidation [61]. In control *ob*/*ob* mice, impaired or inactive AMPK levels were downregulated by MLPH in a dose-dependent manner, subsequently activating the AMPK pathway and resulting in the suppression of cellular lipid accumulation. This is key for proteins that regulate carbohydrate and lipid metabolism [62].

SREBP-1 protein is involved in adipogenesis and increased lipid content in both liver and adipose tissues [63]. SREBP-1 protein overexpression causes fatty liver with hepatic steatosis and triglyceride deposition in the liver, leading to obesity and diabetes [64]. The current results indicate that control obese mice had elevated SREBP protein expression, including fat and triglyceride deposition in liver tissues. SREBP protein expression was downregulated by involving p-AMPK [65]. Western blot analysis suggested that SREBP-1 protein expression was inhibited by MLPH treatment in a dose-dependent manner. Downregulated SREBP1 expression might inhibit lipid accumulation by inactivating SREBP-1 protein expression. The beneficial effect of MLPH is inhibition of SREBP-1 protein expression by activating AMPK phosphorylation. FAS expression is correlated with SREBP-1 expression, causing liver steatosis [66]. Hence, inhibiting FAS expression has been proposed as a strategy to treat obesity, liver steatosis, and diabetes [67]. Acetyl-CoA carboxylase (ACC), including FAS, is a key enzyme for the synthesis of long-chain fatty acids, which can regulate the rate of hepatic triglyceride synthesis. The results show that FAS and ACC levels in control *ob*/*ob* mice were increased compared to the normal group. Thus, these are important contributors to the development of fatty liver [68]. An elevated insulin level is also a major cause of increased FAS and ACC levels in liver tissues [9]. Moreover, the CON *ob*/*ob* group showed increased expression of FAS and ACC without promotion of phosphorylation in the liver. The elevated FAS and ACC levels were downregulated by MLPH in a dose-dependent manner. This trend indirectly supports the hypothesis that MLPH treatment reduces the FAS, ACC and liver steatosis by activation of AMPK phosphorylation in a dose-depended manner. Lee et al. [69] suggested that increased AMPK phosphorylation was observed by treatment with dietary silk peptide in high-fat induced obesity mice. These results indicate that MLPH treatment may be responsible for upregulating several pathways, including lipolysis and fatty acid oxidation.

The increased liver weight, including hepatic steatosis, in control *ob*/*ob* mice might be explained by associated changes in hepatic levels of mRNA, such as those encoding key enzymes of gluconeogenesis (PEPCK and G6Pase), and expression of lipogenic enzymes (FAS, ACC, SCD), including AMPK and SREBP protein expression, which are important factors for lipid synthesis. The expression of hepatic gluconeogenic enzymes PEPCK and G6Pase can lead to chronic hepatic glucose production, hyperglycemia, and insulin resistance in obese and diabetic mice [70]. Our results demonstrated that the mRNA levels of PEPCK and G6Pase mRNA were elevated in CON *ob*/*ob* mice, indicating the production of excess glucose in obese mice. These levels were suppressed in liver tissues in MLPH-treated groups to maintain glucose homeostasis. Glycogen synthesis is regulated by GS and GP activity. Glycogen synthesis was diminished along with the decreasing expression of GS in the presence of lipid accumulation. Elevated lipid levels increase hexosamine flux in adipocytes, which can modulate glycogen synthase [71,72]. These results indicate that the expression of mRNAs encoding lipogenic enzymes FAS, ACC, and SCD was elevated in control obese mice due to inactivated AMPK, leading to the accumulation of fat and liver steatosis. MLPH may activate phosphorylated AMPK signaling and potentially inhibit mRNA expression of FAS, ACC, and SCD transcription factors, leading to a reduction of mRNA transcript and downregulation of enzymatic activity [73,74]. These results indicated that the MLPH fraction has an inhibitory effect on lipogenesis, thus more effectively preventing liver steatosis in *ob*/*ob* mice than other drugs, due to their small peptide sequences with lower molecular weight (≤1 kDa). Fatty acid oxidation or β-oxidation deficiency occurs when mRNA expression of MCAD and CPT is suppressed, leading to fatty liver [75]. In contrast, PDK gene mRNA expression is elevated in obese mice [76]. However, MLPH treatment enhances CPT mRNA expression and prevents lipid accumulation in liver tissues. It can also decrease levels of proinflammatory mediators such as TNF and MCP [77]. Moreover, MLPH can deplete PDK gene expression, leading to reduced fatty acid synthase (FAS) activity in lipid metabolism [78].

In this report, we extracted a short peptide chain (KDLEL) from *M. lusoria* using protamex is a promising method for the recovery of bioactive compounds. This was the first evidence that the production of bioactive peptide (Lys-Asp-Leu-Glu-Leu) effectively suppresses the lipogenic protein (SREBP, FAS, and ACC) and gene expressions, and down-regulates obesity and diabetes. The peptide sequence mainly comprised hydrophobic amino acids (leucine), which have a greater free radical scavenging effect and ability to effectively interact with lipid materials. Moreover, the MLPH fraction effectively suppresses free radical toxicity and enhances antioxidant enzymes activities. Based on these results, we propose that *M. lusoria* hydrolysate fractions have a beneficial effect on obesity and diabetes. This is the first report on the anti-obesity and anti-hyperglycemic effects of protamex hydrolysate possessing antioxidant peptide from *M. lusoria* in *ob*/*ob* mice. However, this was a preclinical study using an animal model in laboratory conditions, which is an important limitation. These bioactive peptides and formulations must be stabilized to extend their shelf-life to obtain the same effect in clinical studies.

## 4. Materials and Methods

### 4.1. Preparation of Protein Hydrolysate Fractions from M. lusoria

In this experiment, *M. lusoria* was purchased from Yeosu Fish Market. Protein hydrolysate fractions were prepared using neutrase, protamex, alcalase and flavourzyme enzymes under the optimal conditions for each enzyme. In this process, *M. lusoria* dry powder (50 g) and enzyme (0.5 g) were mixed (1:100 (*w*/*w*) enzyme/substrate ratio). After adjusting the pH to 6.0 at 50 °C for neutrase, 6.0 at 60 °C for protamex, 8.0 at 50 °C for alcalase, and 7.0 at 50 °C for flavourzyme, the hydrolysis process was carried out with continuous agitation for 24 h. For enzyme inactivation, the total mixture was then heated for 10 min at 100 °C in a water bath. Unhydrolyzed protein molecules were removed by a filtering process using a filter cloth. The extraction yield was calculated as the percentage of the weight of enzymatic hydrolysate to the dried weight of *M. lusoria* protein content used for extraction. The filtrate sample was fractionated through the Quixstand benchtop system (GE Healthcare, Buckinghamshire, UK) using an ultrafiltration membrane, producing four fractions: ≥5 kDa, 3–5 kDa, 1–3 kDa and ≤1 kDa according to the previous method [79]. The MLPH ≤ 1 kDa fraction was freeze-dried and used for further studies.

### 4.2. Antioxidant Activity of M. lusoria Hydrolysate Fractions

The antioxidant activity of *M. lusoria* hydrolysate fractions was assessed using DPPH and ABTS radicals in vitro. The scavenging methods of both radicals were modified and an analysis was performed with 96-well plates using three replicates. The DPPH radical scavenging activity analysis method was conducted as described previously [80]. Briefly, a methanolic 0.208 mM DPPH solution was prepared, along with different concentrations of extract sample (0.0625, 0.125, 0.25, 0.5, 1, 2, and 4 mg/mL). Then DPPH radical solution (150 µL) was mixed with 50 µL of each sample concentration. The 96-well plate was kept in a dark place for 20 min at room temperature, then absorbance at 540 nm was recorded. The DPPH radical inhibition percentage (%) was calculated using the following formula: radical scavenging activity (RSA) = 100 × (A_control_ − A_sample_)/A_control_, where A_control_ and A_sample_ are absorbance values. Half inhibitory concentration (IC_50_) values were also calculated using the RSA relationship curve with fraction concentrations of respective samples.

An ABTS radical scavenging test was performed using a previously described method [81] with slight modifications. The scavenging activity value was calculated using the following equation: % inhibition = 100 (A_control_ − A_sample_)/A_control_. IC_50_ was calculated from the radical scavenging activity percentage (%) versus the respective sample concentration curve. To determine scavenging activity, the ABTS reagent was prepared by mixing 7 mM of ABTS with potassium persulfate (140 mM) and the mixture solution was kept at room temperature in a dark place for 16 h to induce free radical formation, then diluted with water. For the analysis, 100 µL of the extracted sample was mixed with 100 µL of ABTS reagent in a 96-well microplate. After incubation for 6 min at room temperature, absorbance was measured at 734 nm. For this process, 100% methanol was used as a control. The following equation was used: ABTS radical scavenging activity (%) = (Blank OD − Sample OD)/Blank OD × 100.

### 4.3. Purification and Identification of MLPH Peptide Fraction Using LC-MS/MS Analysis

The MLPH fraction was dissolved using distilled water (DW) and sub-fractions were collected using a Sephadex G-25 gel filtration column (2.5 × 70 cm) (GE Healthcare, Chicago, IL, USA), which was previously equilibrated with DW. Four sub-fractions were collected after eluting the column with DW at a flow rate of 1.5 mL/min (fraction volume 7.5 mL). The sub-fraction scavenging activity was measured using DPPH radicals. All sub-fractions were injected into a Dionex Ultimate 3000 preparative reversed-phase high performance liquid chromatography (RP-HPLC) system (Thermo Scientific Inc., Dionex, Sunnyvale, CA, USA) equipped with a C18 analytical column (YMC-Triart C18 ExRs, 250 × 4.6 mm, 5 µm particle size; YMC Co., Ltd., Kyoto, Japan). The samples were detected at 254 and 280 nm wavelengths by injecting 20 μL of MLPH sub-fractions 1–4. The mobile phase for the analysis was distilled water and MeOH containing 0.1% formic acid, flowing from 10–100% methanol (*v*/*v*) at 1 mL/min flow rate for 40 min under a gradient system. Following the highest yield and activity analysis, the most potent peptide sub-fraction was purified by RP-HPLC on a C18 analytical column (YMC-Triart C18 ExRs) with the same MeOH containing 0.1% formic acid at a flow rate of 1 mL/min for 40 min [30].

Finally, the sub-fraction amino acid sequence was analyzed using a Micro Q-TOF III ESI mass spectrometer (Bruker Daltonics, Bremen, Germany) equipped with an electrospray ionization (ESI) source. The instruments were in positive-ion mode and calibrated with the Tunemix™ mixture (Agilent Technologies, Palo Alto, CA, USA). The ion source was operated in positive mode with capillary V (+): 4000 V, nitrogen gas used for drying; flow rate: 5.5 L/min, temperature: 180 °C; scan range: 50–2000 *m*/*z* with mass accuracy was better than 5 ppm; collision energy: 7 eV using argon as collision gas [82].

### 4.4. Animals

As experimental animals, five-week-old male C57BL/6J mice (22–23 g body weight (BW)) and *ob*/*ob* mice (37–38 g BW) were obtained from Orient Bio (Seongnam, Korea). The animals were housed according to the Chonnam National University Guidelines for Care and Use of Laboratory Animals. They were acclimated in a room at 22 ± 2 °C with humidity controlled at 55 ± 5% over a 12 h light/dark cycle for one week before the experiments.

### 4.5. Experimental Design

In this experiment, C57BL/6J mice were classified as the normal (NOR) group and *ob*/*ob* mice were categorized into three groups (*n* = 6): six mice in the control (CON) group and six mice each receiving *M. lusoria* protamex hydrolysate at low (MLPH-L) or high (MLPH-H) concentration. All mice were maintained for six weeks with feed containing the following ingredients per 1 kg diet: sucrose (500 g), casein lactic acid (200 g), cellulose (50 g), corn starch (150 g), corn oil (50 g), mineral mix (35 g), vitamin mix (10 g), choline bitartrate (2 g), and DL-methionine (3 g). Animals in the normal and control groups were administered 1 mL of distilled water (DW) orally. Other groups were orally administered 1 mL DW containing MLPH once a day (09:00–10:00) for six weeks. The hydrolysate dose was 150 mg/kg BW in the MLPH-L group and 300 mg/kg BW in the MLPH-H group. Body weight changes, dietary intake, and water intake were measured daily (08:00–09:00) during the experimental period. The food efficiency ratio (FER) was calculated by dividing body weight gain during the total experiment period (g/42 days) by daily food intake (g/day).

Mice were anesthetized using isoflurane (Hana Pharm Co., Ltd., Seongnamm, Korea) via inhalation after 6 weeks. Blood samples were collected into heparin tubes and centrifuged at 2000× *g* for 10 min at 4 °C to obtain serum samples for determining biochemical parameters. Organs were isolated, washed with physiological saline, and weighed after removing moisture. They were quickly frozen with liquid nitrogen and stored at –80 °C for analysis. Fat was collected from different regions and weighed. Liver and fat epidermal tissues were fixed with 10% formalin and used for histological analysis. Frozen liver tissues were homogenized with 450 mM phosphate buffer (pH 7) at a ratio of 1:9 using a homogenizer and centrifuged for 20 min at 25,000× *g* rpm at 4 °C. The supernatant was collected and used for the analysis of lipid and antioxidant activity. The experimental protocols used in this study were approved by the Animal Ethics Committee of Chonnam National University (No. CNU IACUC-YS-2020-9).

### 4.6. Glucose Tolerance Test

For the glucose tolerance experiment, *ob*/*ob* mice fasted 1 day after the 6th week of the experiment, and 2 g/kg body weight of glucose was injected intraperitoneally. A glucose tolerance test was performed after measuring final fasting blood glucose levels. Blood samples were collected from tail veins of normal and experimental *ob*/*ob* mice at 0, 30, 60, 90, 120, and 180 min. Changes in blood glucose levels in different groups were confirmed using a glucose meter (Accu-Chek Performance Kit, Roche Diagnostics Korea Co., Ltd., Seoul, Korea) [17].

### 4.7. Serum Biochemical Analysis

TNF-α, MCP-1, insulin (INS), and adiponectin were measured using an ELISA kit (Elabscience Inc., Houston, TX, USA). TBARS levels were measured using a colorimetric assay kit (Elabscience, Inc., Houston, TX, USA) [83]. Glutamic oxaloacetic transaminase (AST) and glutamic pyruvic transaminase (ALT) activity and total cholesterol (TC), LDL cholesterol, HDL cholesterol, and triglyceride (TG) levels were determined using enzymatic analysis kits (Asan Pharmaceuticals, Hwasung, Korea) [84]. Total protein (Biuret test kit), albumin (BCG kit), blood urine nitrogen (BUN) (urease GLDH test kit), and creatinine were analyzed using IDMS Traceable test and an automatic blood analyzer (Cobas C702, Roche Diagnostics) [85].

### 4.8. Total Cholesterol and Triglyceride Levels in Liver Tissues

TC and TG levels in liver tissues were measured using a chemistry analyzer (AU480, Beckman Coulter, Inc., Brea, CA, USA) according to the previous method [86].

### 4.9. Antioxidant Enzyme Activity in Liver Tissues

Liver tissue SOD, CAT, GSH, GPX, GR, and GST activities were determined using a colorimetric assay kit (Biovision Inc., San Francisco, CA, USA) following the manufacturer’s instructions [87].

### 4.10. Liver Tissue Extracts and Western Blotting

Isolation of proteins and western blotting analysis was conducted according to previously reported methods [88] with slight modifications. In this process, liver tissues were homogenized with a homogenizer (Polytron CH-6010, Kinematica GmbH, Luzern, Switzerland) using buffer (50 mM Tris, 0.1% sodium dodecyl sulfate (SDS), 150 mM sodium chloride, 1% Triton X-100, 1% sodium deoxycholate, 1 mM EDTA, and 1 mM sodium orthovanadate) at 4 °C. The homogenate was obtained and centrifuged at 12,000× *g* rpm for 20 min at 4 °C (MX-160, Tomy Seiko Co., Ltd., Tokyo, Japan). The final supernatant was retained as total cell lysate. The obtained residual part was resuspended in buffer (0.5 mL) and used as a membrane fraction. Then 100 μg of protein containing liver homogenate was subjected to Western blotting for AMPK, SREBP, FAS, and ACC as described previously [89].

### 4.11. Isolation and Quantification of RNA Using Real-Time RT-PCR

Total RNA was collected from liver tissue using TRIzol RNA isolation reagent (Invitrogen, Carlsbad, CA, USA). RNA reverse transcription was carried out using Prime Script^TM^ 1st Strand cDNA Synthesis Kit (Takara Bio Inc., Shiga, Japan) according to the manufacturer’s instructions. A quantitative RT-PCR reaction was conducted using 20 μL SYBR^®^ Premix Ex Taq (Takara Bio Inc. Kusatsu, Shiga, Japan). The result was normalized against the β-actin mRNA signal. The primer sequences used in this study are listed in Table 4 [17].

### 4.12. Hematoxylin and Eosin (H&E) Staining for Histological Analysis

For histological analysis, tissues were fixed with formalin (10% (*v*/*v*)) using phosphate buffer, and then embedded with paraffin wax. Next, 4–6 µm thick sections were prepared, and each section was stained with H&E stain. The histological changes were examined under an optical microscope integrated with a camera (Olympus DP70, Olympus Optical Co., Tokyo, Japan), and fat deposition and area of the adipocyte in epididymal fat tissue were analyzed [90]. The length of the adipocyte cell in fat tissue could be measured in the H&E staining images using SABIA (Solution for Automatic Bio-Image Analysis) software (EBIOGEN, Seoul, Korea).

### 4.13. Statistical Analysis

Data are expressed as means ± SEM. All statistical analyses were carried out using the IBM SPSS statistic version 20 (Statistical Package for Social, SPSS Inc., Chicago IL, USA). The data were evaluated by one-way analysis of variance (ANOVA) followed by Tukey’s multiple comparison test. Differences were considered significant at *p* < 0.05.

## 5. Conclusions

In the present study, we purified and identified a pentapeptide (Lys-Asp-Leu-Glu-Leu, 617.35 Da) from MLPH. The MLPH exhibited antioxidant activity based on DPPH radical scavenging assay. Our results confirmed that the treatment of MLPH possessing an antioxidant peptide sequence markedly decreased the levels of blood glucose, serum lipid level, and accumulation of fats in liver and adipose tissues in *ob*/*ob* mice. In addition, MLPH treatment significantly improved impaired glucose tolerance as well as hepatic mRNA expression of gluconeogenesis and lipogenesis-related enzymes in *ob*/*ob* mice. These findings suggest that MLPH has a positive impact on obesity-induced diabetes mice, and could be used as a functional food ingredient for human health. Further investigation is needed to obtain additional bioactive peptides from *M. lusoria* and improve their stability by applying novel formulation methods. In addition, clinical trials are needed to determine their beneficial effects on obesity, diabetes, and other medical conditions.

## Figures and Tables

**Table 1:** Enzymatic hydrolysate yield (%) and molecular weights of fractions (kDa) with IC_50_ values (mg/mL) indicate ABTS and DPPH radical scavenging activity (%).

Sample Name	Yield (%)	Enzymatic Hydrolysate Fractions							
		DPPH Radical IC_50_ Value (mg/mL)				ABTS Radical IC_50_ Value (mg/mL)			
		≤1 kDa	1–3 kDa	3–5 kDa	≥5 kDa	≤1 kDa	1–3 kDa	3–5 kDa	≥5 kDa
Neutrase	65.21	1.38	2.97	2.43	2.21	2.67	3.30	3.21	3.10
Protamex	64.08	1.38	2.93	2.10	1.63	1.58	1.51	1.71	1.50
Alcalase	59.73	1.34	2.07	2.48	2.20	1.27	1.56	1.53	1.56
Flavourzyme	59.89	1.28	2.77	1.80	1.82	1.50	1.48	1.80	1.82

IC_50_: Half maximal inhibitory concentration. DPPH: 2,2-Diphenyl-1-picrylhydrazyl, and ABTS: 2,2′-azino-bis (3-ethylbenzothiazoline-6-sulfonic acid).

**Table 2:** Food efficiency ratio (FER), body weight, organ weight, and fat content in NOR, CON, and MLPH groups after treatment with protamex extract at a low or high dose for 6 weeks in *ob*/*ob* mice fed a standard diet.

Measurement	NOR	CON	MLPH-L	MLPH-H
Initial body weight (g)	22.33 ± 0.33 ^b^	38.33 ± 0.88 ^a^	38.67 ± 1.33 ^a^	37.67 ± 1.67 ^a^
Water intake (mL/day)	3.21 ± 0.03 ^b^	6.63 ± 0.09 ^a^	6.47 ± 0.61 ^a^	6.24 ± 0.19 ^a^
Food intake (g/day)	3.16 ± 0.027 ^b^	3.91 ± 0.077 ^a^	4.12 ± 0.140 ^a^	3.99 ± 0.035 ^a^
Food efficiency ratio (FER) (g)	1.48 ± 0.10 ^b^	2.47 ± 0.12 ^a^	2.60 ± 0.34 ^a^	2.83 ± 0.31 ^a^
Body weight gain (g/42 days)	4.67 ± 0.33 ^b^	9.67 ± 0.33 ^a^	10.67 ± 1.20 ^a^	11.33 ± 1.33 ^a^
Heart (g)	0.128 ± 0.001 ^b^	0.155 ± 0.009 ^a^	0.142 ± 0.007 ^ab^	0.136 ± 0.004 ^ab^
Kidney (g)	0.322 ± 0.002 ^b^	0.365 ± 0.011 ^a^	0.343 ± 0.008 ^ab^	0.323 ± 0.013 ^b^
Liver (g)	1.433 ± 0.021 ^c^	3.582 ± 0.007 ^a^	3.285 ± 0.052 ^b^	3.248 ± 0.093 ^b^
Gastrocnemius muscle (g)	0.170 ± 0.004 ^NS^	0.163 ± 0.010	0.174 ± 0.011	0.184 ± 0.009
Fat mesenteric (g)	0.552 ± 0.006 ^c^	1.733 ± 0.015 ^a^	1.307 ± 0.055 ^b^	1.219 ± 0.090 ^b^
Fat retroperitoneal (g)	0.881 ± 0.010 ^b^	3.109 ± 0.031 ^a^	2.916 ± 0.152 ^a^	2.997 ± 0.063 ^a^
Fat epididymal (g)	0.013 ± 0.0004 ^c^	0.027 ± 0.0023 ^a^	0.020 ± 0.0005 ^b^	0.019 ± 0.0010 ^b^
Fat kidney (g)	0.083 ± 0.004 ^c^	0.683 ± 0.050 ^a^	0.417 ± 0.056 ^b^	0.395 ± 0.010 ^b^
Fat total (g)	1.529 ± 0.008 ^c^	5.553 ± 0.079 ^a^	4.659 ± 0.106 ^b^	4.630 ± 0.108 ^b^

Each value represents the mean ± SEM (*n* = 6). ^a–c^ Values not sharing a common letter are significantly different at *p* < 0.05 by Tukey’s multiple comparison test, ^NS^: not significant.

**Table 3:** Effects of 6 week treatment with low and high dose MLPH extract on hepatic antioxidant enzymes in *ob*/*ob* mice fed a standard diet.

Liver Analysis	NOR	CON	MLPH-L	MLPH-H
GSH (nmol/mg)	456.16 ± 9.06 ^a^	202.26 ± 3.19 ^d^	259.45 ± 14.58 ^c^	335.46 ± 0.86 ^b^
GST activity (U/mL)	3.49 ± 0.38 ^a^	1.96 ± 0.21 ^b^	1.58 ± 0.36 ^b^	1.72 ± 0.19 ^b^
GPx activity (U/mL)	1.63 ± 0.35 ^c^	0.71 ± 0.10 ^d^	2.48 ± 0.23 ^b^	3.27 ± 0.38 ^a^
GR activity (mU/mL)	22.57 ± 0.95 ^a^	16.28 ± 3.14 ^b^	17.36 ± 1.28 ^b^	19.61 ± 0.64 ^ab^
SOD activity (U/mL)	3.08 ± 0.01 ^a^	2.69 ± 0.01 ^d^	2.86 ± 0.01 ^c^	3.02 ± 0.02 ^b^
CAT colorimetric activity (U/mL)	1.668 ± 0.007 ^b^	1.662 ± 0.001 ^b^	1.670 ± 0.004 ^b^	1.685 ± 0.001 ^a^

Glutathione (GSH), glutathione S-transferase (GST), glutathione peroxidase (GPx), glutathione reductase (GR), Superoxide dismutase (SOD), catalase (CAT). Each value represents the mean ± SEM (*n* = 6). ^a–c^ Values not sharing a common letter are significantly different at *p* < 0.05 by Tukey’s multiple comparison test.

**Table 4:** List of primers used in this study for gene expression analysis.

Gene	Forward Primer	Reverse Primer
PEPCK	CTGGCACCTCAGTGAAGACA	TCGATGCCTTCCCAGTAAAC
G6Pase	ATGACTTTGGGATCCAGTCG	TGGAACCAGATGGGAAAGAG
GS	GACACTGAGCAGGGCTTTTC	GGGCCTGGGATACTTAAAGC
LGP	CCAGAGTGCTCTACCCCAAT	CCACAAAGTACTCCTGTTTCAGC
FAS	CCCTTGATGAAGAGGGATCA	ACTCCACAGGTGGGAACAAG
ACC	GACGTTCGCCATAACCAAGT	CTGCAGGTTCTCAATGCAAA
SCD	AGCTGGTGATGTTCCAGAGG	GTGGGCAGGATGAAGCAC
MCAD	AGCTGATTGGCAATGTCTCCAGCAAA	GATCGCAATGGGTGCTTTTGATAGAA
PDK	ATCTAACATCGCCAGAATTAAACC	GGAACGTACACAATGTGGATTG
CPT	GAGCCACGAAGCCCTCAAACACAT	GCTGTACAACATGGGCTTCCGACCTG

PEPCK, phosphoenolpyruvate carboxykinase; G6Pase, glucose 6-phosphatase; GS, glycogen synthase; LGP, liver glycogen phosphorylase; FAS, fatty acid synthase; ACC, acetyl-CoA carboxylase; SCD, stearoyl-CoA desaturase; MCAD, medium chain acyl-CoA dehydrogenase; CPT, carnitine palmitoyltransferase; PDK, pyruvate dehydrogenase kinase.

## Data Availability

The data of the research were not shared.

## Supplementary Materials

The following supporting information can be downloaded at: https://www.mdpi.com/article/10.3390/ijms23074015/s1.
