# Supplementary material for: Anti-Obesity and Anti-Hyperglycemic Effects of Meretrix lusoria Protamex Hydrolysate in ob/ob Mice
Source: Int J Mol Sci. 2022 Apr 5;23(7):4015. doi: 10.3390/ijms23074015 (PMC8999646; doi:10.3390/ijms23074015)
Supplement: Supplementary file 1 [file ijms-23-04015-s001.zip › ijms-1663214-supplementary.pdf]

Supplementary Material

Anti-obesity and Anti-hyperglycemic Effects of *Meretrix lusoria* Protamex Hydrolysate in *ob/ob* mice

Min Ju Kim [1,†], Ramakrishna Chilakala [1,†], Hee Geun Jo [1], Seung-Jae Lee [2], Dong-Sung Lee [3], Sun Hee Cheong [1,*]

[1] Department of Marine Bio-Food Sciences, College of Fisheries and Ocean Sciences, Chonnam National University, Yeosu 59626, Korea.
modori96k@naver.com (M.J.K.); ramach2006@gmail.com (R.C.); altkwh@naver.com (H.G.J.)
[2] Immunoregulatory Material Research Center, Korea Research Institute of Bioscience and Biotechnology (KRIBB), Jeongeup 56212, Korea; seung99@kribb.re.kr
[3] Department of Pharmacy, College of Pharmacy, Chosun University, Dong-gu, Gwangju, 61452, Korea
dslee2771@chosun.ac.kr

* Correspondence: sunny3843@jnu.ac.kr; Tel.: (Phone: +82-61-659-7215; Fax: +82-61-659-7219)

† These authors contributed equally to this study.

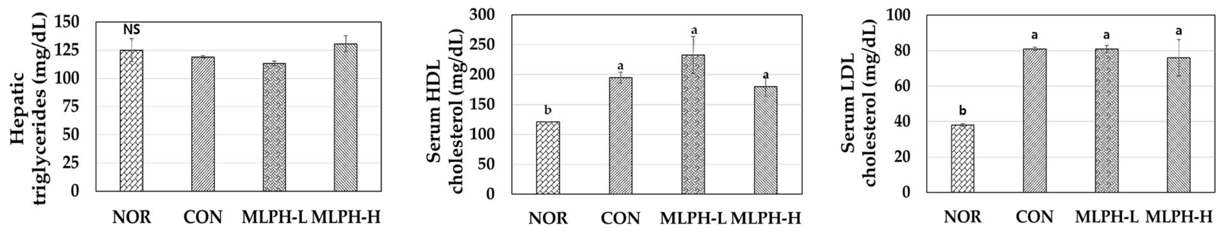

(A) (B) (C)

Figure S1. Effect of MLPH fraction on (A) hepatic triglycerides; (B) serum HDL cholesterol, and (C) serum LDL cholesterol. Each value represents the mean ± SEM (n=6). [a-b] Values not sharing a common letter are significantly different at $p < 0.05$ by Tukey's multiple comparison test, [NS] : not significant.

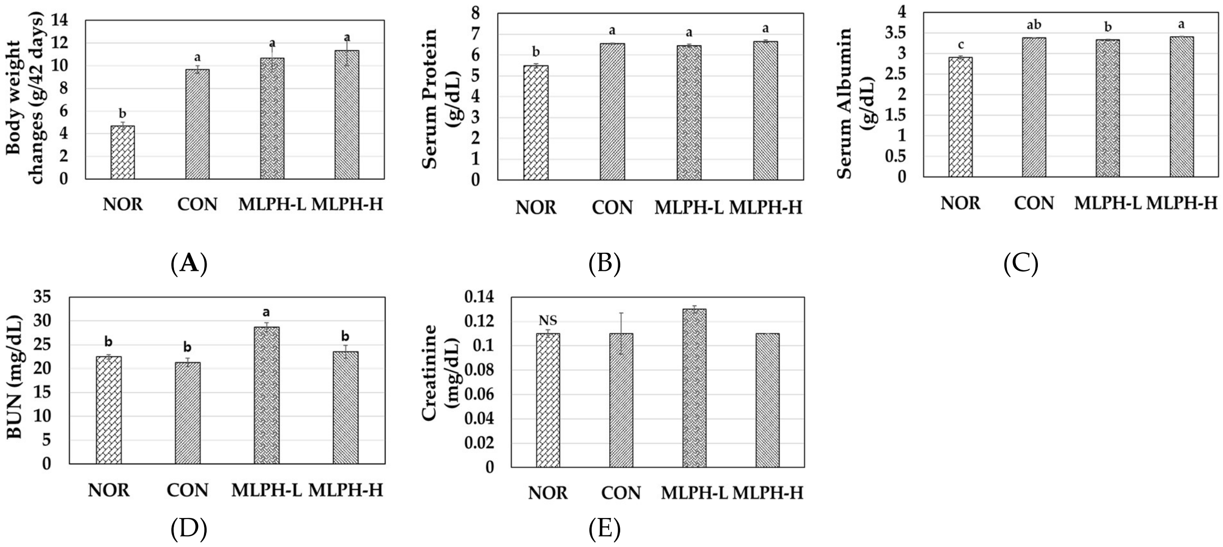

Figure S2. Effects of MLPH fraction on (A) mouse body weight changes during 6 weeks of treatment; metabolic parameters: (B) serum protein, (C) serum albumin, (D) blood urea nitrogen (BUN), and (E) creatinine. Each value represents the mean ± SEM (n=6). [a-c] Values not sharing a common letter are significantly different at $p < 0.05$ by Tukey's multiple comparison test, [NS] : not significant.
